# Changes in the Electrical Activity of the Brain in the Alpha and Theta Bands during Prayer and Meditation

**DOI:** 10.3390/ijerph17249567

**Published:** 2020-12-21

**Authors:** Paweł Dobrakowski, Michal Blaszkiewicz, Sebastian Skalski

**Affiliations:** 1 Institute of Psychology, Humanitas University, 41-200 Sosnowiec, Poland; michal.blaszkiewicz@poczta.fm; 2 Institute of Psychology, Polish Academy of Sciences, 00-378 Warsaw, Poland; sebastian.skalski@sd.psych.pan.pl

**Keywords:** meditation, prayer, EEG, alpha band, theta band

## Abstract

Focused attention meditation (FAM) is a category of meditation based on an EEG pattern, which helps the wandering mind to focus on a particular object. It seems that prayer may, in certain respects, be similar to FAM. It is believed that emotional experience correlates mainly with theta, but also with selective alpha, with internalized attention correlating mainly with the synchronous activity of theta and alpha. The vast majority of studies indicate a possible impact of transcendence in meditation on the alpha wave in EEG. No such reports are available for prayer. Seventeen women and nineteen men aged 27–64 years with at least five years of intensive meditation/prayer experience were recruited to participate in the study. We identified the two largest groups which remained in the meditation trend originating from the Buddhist system (14 people) (Buddhist meditators) and in the Christian-based faith (15 people) (Christian meditators). EEG signal was recorded with open eyes, closed eyes, during meditation/prayer, and relaxation. After the EEG recording, an examination was conducted using the Scale of Spiritual Transcendence. Buddhist meditators exhibited a statistically significantly higher theta amplitude at Cz during meditation compared to relaxation. Meanwhile, spiritual openness favored a higher theta amplitude at Pz during relaxation. Our study did not reveal statistically significant differences in frontal areas with regard to alpha and theta, which was often indicated in previous studies. It seems necessary to analyze more closely the midline activity in terms of dispersed neural activity integration.

## 1. Introduction

Spirituality plays an important role in maintaining health understood as full well-being. As recently as a dozen or so years ago in Poland, it was almost solely identified with Catholic religiousness. However, there have been major changes in this respect. Religion is an extremely difficult subject to research, not least because it belongs to a very sensitive and intimate sphere of individuals’ and communities’ lives—religion, faith, and religiousness. Many aspects of religiosity are not subject to statistical research, while some of them escape any empirical studies at all, e.g., the phenomenon of “non-institutional religiousness”. It seemed interesting to us whether these various forms of spirituality have a different impact on the electrophysiological activity of the brain. We wondered whether the possible effect would be more pronounced in people with a higher level of spiritual transcendence.

Meditation is a practice of self-regulation that improves mental and emotional control [1]. We already have some knowledge about whether meditation can change the electrophysiological activity of the brain [2]. Meditation practices may be divided into three main categories: open monitoring meditation (OMM), focused attention meditation (FAM), and transcendental meditation (TM) [3].

FAM is usually a starting point for each novice meditator. During FAM, practitioners are expected to turn their attention to a selected object or event such as breathing, mantra recital, or visualization. In order to maintain that focus, the practicing person needs to constantly monitor their concentration on a selected event in order to prevent the mind from wandering [4,5]. FAM is mostly identified by inducing gamma power during meditation. The frequency of the gamma band is used most often to study the process of learning, as well as cognitive processing—including attention and working memory [2].

Initial research on meditation focused mainly on alpha activity, while significant changes occur also in theta activity, which made some researchers suggest that theta is the main brain activity specific to the state of meditation. Aftanas and Golocheikine believe that emotional experience correlates mainly with theta, but also with selective alpha, with internalized attention correlating mainly with the synchronous activity of theta and alpha [6].

Takahashi et al. observed that during meditation there is an increase in theta high-frequency power and the low alpha frequency in the anterior cerebral regions, as well as a decrease in the activity of the sympathetic system and an increase in the activity of the parasympathetic system. The author stresses that theta and alpha are independently involved in thought processes during meditation. The researcher also suggests that effective meditation leads to slowed down synchronous alpha activity in the frontal areas combined with a decrease in the activity of the sympathetic system which, in turn, may lead to the occurrence of theta in the frontal areas and the increase of activity of the parasympathetic system [7].

So far, Christian prayer has very rarely been the subject of EEG examination. Christian prayer covers a wide range of cognitive processes, including attention, memory, decision making, and planning [8]. It seems that prayer may, in certain respects, be similar to FAM [3,9].

Faber also suggests that, despite clear differences in the religious technique and orientation of Buddhist meditators and those undertaking prayer, it seems there are significant similarities in the neural correlates of spiritual love (love generated during a loving kindness meditation performed by Buddhist meditators, and love generated during prayer) [10]. Neurophysiological fundamentals and the results of previous studies indicate higher brain activity in the beta2 and gamma frequencies during prayer [3,11]. However, these ranges are relatively difficult to monitor in the EEG due to the ease of overlap between muscle artifacts in these bands [12]. Furthermore, it seems that people who pray on a regular basis may be more skillful in absorbing incoming information and in avoiding task-irrelevant thoughts [13].

Lagopoulos et al. monitored changes in the alpha and theta bands, comparing non-directive meditation (open monitoring) with complete relaxation. The team observed a significant increase in the theta power for the state of meditation, especially in the frontal and temporal-central regions [14]. We decided to monitor the alpha and theta bands during prayer/meditation, as well as compare them with the electrophysiological activity during a cognitively engaging task. This seems to be reasonable since different neural networks operate in parallel when processing information [15]. Low-frequency rhythms (theta and alpha) reflect top-down information processing involving attention and reduced working memory activity [16].

Currently, four methods may be commonly used to collect data regarding cerebral activity: EEG, NIRS, fMRI, and PET [17]. Of the four, EEG seems to be the best compromise between high temporal resolution, availability, and cost of the study. Magnetoencephalography (MEG) is also worth noting, as it delivers more precise data than EEG. However, this method is limited by high costs and the difficulty to obtain high-temperature superconductivity. As for invasive methods such as EcoG, these were not taken into account due to the nature of the study.

A balance in EEG alpha and theta activity along the anterior-posterior axis is implicated in the construct of transient prefrontal cortex deregulation. Relative to a resting wakefulness baseline, the frontal lobe becomes less active (hence, more alpha activity), and is balanced by a greater degree of parietal activation. If the frontal lobe becomes less active, then cognitive functions such as planning and decision making (the executive functions) become impaired. A similar impairment would occur to reflective awareness, as the individual’s capacity to engage in reflective thinking is reduced [18]. Brain oscillations at alpha and theta frequency bands have been shown to play a key role in a wide variety of cognitive tasks involving memory and executive control. While alpha oscillations have been associated with the storage and retrieval of information, theta oscillations have been associated with the manipulation of information [19].

### 1.1. Alpha

A power increase in the alpha band (8–12 Hz) has been considered a sign of cortical idling [20].

The alpha band has been used as a sign of relaxation during meditation and other types of rest. The number of alpha waves increase when the brain relaxes from intentional, goal-oriented tasks. This is a sign of deep relaxation, but it does not mean that the mind is void [14].

### 1.2. Theta

The activity of the theta band (4–8 Hz) in the medial frontal and anterior cingulate cortex is a neuronal indicator for monitoring internal processes and cognitive control [21]. For this reason, we expect theta to increase in this area during meditation, which involves monitoring the ongoing experience without a high level of control or manipulation of the experience content. We may have similar expectations with regard to prayer.

### 1.3. Transcendence

The transcendent state is described as one of relaxed wakefulness in a phenomenologically different space–time. In different cultures, transcendent states achieved through meditation practices have been recorded. Transcendent states are experienced as a continuity of awareness despite the absence of sensory or cognitive perception. Practices of reaching a state of transcendence vary from meditation through yoga to contemplative prayer. People who experience transcendence describe a similar unifying, ineffable state of consciousness [22]. 

A change in reflective awareness characterizes the transition from trance to transcendence. There seems to be an accompanying shift in the electrophysiological state centered on the predominance of frontal-midline theta activity, as well as a change in posterior alpha asymmetry. While the study of mind and behavior can progress without taking into consideration the ongoing cortical activity, it is an appreciation of the changing dynamics along the anterior-posterior axis, with its electrophysiological signature of alpha-theta power [18].

The authors understand spiritual transcendence as “the capacity of individuals to stand outside of their immediate sense of time and place and to view life from a larger, more objective perspective” [23].

## 2. Materials and Method

### 2.1. Characteristics of the Test Group

Seventeen women and nineteen men aged 27–64 years with at least five years of intensive meditation/prayer experience were recruited to participate in the study. Study volunteers were recruited from meditation/prayer groups. Each of them declared at least 2 years of intensive experience with regard to prayer/meditation.

Prior to the commencement of the study, the entire procedure had been thoroughly explained to the participants. They were also informed that the purpose of the study was to record their EEG activity in different situations. All participants offered their written, voluntary consent to participate in the study. Subjects reported no neurological or psychological diseases and did not take drugs affecting the central nervous system.

We then asked about the employed prayer/meditation techniques and the trend from which they originated. This allowed us to identify the two largest groups which remained in the meditation trend originating from the Buddhist system (14 people) (Buddhist meditators) and in the Christian prayer trend (15 people) (Christian meditators). 

This study was approved by The Committee for Ethics in Scientific Research of The Institute of Psychology, Polish Academy of Science (IP.403.31.2020).

### 2.2. Test Procedure

The tests were conducted at the Electrophysiological Research Laboratory of the Academy of Physical Education in Katowice. The lab room is well isolated from external disturbing conditions (sound insulation, dimmed lighting) and electromagnetically shielded. The tests were conducted in the evening. Participants were asked to refrain from caffeine consumption and smoking for a day, as well as take care of the quality of their sleep in order to exclude the impact of those factors on the recording [24].

After the initial interview, the participants would sit in a comfortable chair. An EEG cap with 19 electrodes (24-channel Deymed TruScan apparatus, sampling frequency 1024/second) was applied to their heads. The electrodes were placed at points selected as per the 10–20 system. Electrode impedance was kept below 5 kOhm. A 50 Hz line filter, as well as a high-pass and low-pass analog filter (1 and 40 Hz, respectively), were used. The reference electrode was placed on an earlobe.

Manual analysis was performed of the recording and it was divided into 3-s fragments. Those with artifacts were removed and it was ensured that each stage contained at least 20 artifact-free parts. The mean result from those left was subjected to fast Fourier transform using TruScan Explorer (Deymed Diagnostic).

For the alpha band, we investigated occipital electrodes at sites where the alpha activity is usually more pronounced. The crossings between the eyes open and eyes closed spectra provide the frequency boundaries: Lower Transition Frequency and Higher Transition Frequency. The band between them was determined as alpha. The range below 4 Hz was determined as theta.

The analysis was performed by an experienced electroencephalography specialist with an EEG license from the Polish Society of Clinical Neurophysiology.

Detailed analysis of the electrical activity of the brain was performed in the frontal lobe at sites Fp1 (left prefrontal area), Fp2 (right prefrontal area), and Fz (medial frontal area), in the central part of the head at site Cz (central area above the central sulcus), in the parietal lobe at site Pz (medial area) and in the occipital lobe at sites O1 (left area) and O2 (right area). These areas were selected as a central point of clusters suggested by Aftanas [6]. The remaining points were observed in terms of possible epileptiform discharges.

Experiment procedure

1. Collecting a detailed interview and explaining the course of the experiment.
2. Three minutes of EEG signal recording with open eyes. The patient was instructed to think about the “here and now”.
3. Three minutes of EEG signal recording with eyes closed. The patient was instructed to think positively about what will matter-of-factly happen in their lives in the near future, what they are waiting for, and what they want to happen. After these stages, one of the participants was excluded due to an epileptiform discharge
4. Meditation/prayer stage with closed eyes. Patients declared how much time they needed. This was usually around 20–25 min. During this sequence, individuals were left alone, because the person administering the test would leave the room after the start of the recording and would return after an agreed amount of time. Both groups of participants indicated the start and finish of their practices by pressing a button to enable precise data extraction. The recording of this sequence was checked for artifacts, and the results in which the artifacts significantly distorted the EEG image were eliminated from the analysis. Six recordings were rejected at this stage.
5. Relaxation stage, which each time took place under similar conditions, preceded by meditation/prayer (position, closing of eyes). This part lasted 5 min and started when the subject expressed their readiness (on average about 3 min after the end of the meditation/prayer). During this phase, the test facilitator also left the room and signaled its completion after 5 min by quietly knocking on the door three times. Before leaving the room, the subject was instructed to think pleasant thoughts about the nice things that they experienced during their lives. A suggestion was made to recall pleasant events from one’s past. This method of inducing relaxation was specially selected for the study cohort due to the fact that performing any relaxation techniques associated with auto suggestion and autogenic or breathing-related techniques, as declared by the participants, could result in them re-entering a state of meditation/relaxation, in which working with breathing is mostly the basis and starting point for meditation/prayer. Moreover, the choice of this form of relaxation was also dictated by the assumption that it should have an active rather than resting form and it should contain an element of attentiveness and concentration [25].

After analyzing all the data, the records of 10 Subjects belonging to the Buddhist group and 12 Christian meditators could be subjected to further analysis.

6. After the EEG recording, an examination was conducted using the Scale of Spiritual Transcendence [26]. This tool consists of two subscales: transcendence proper (α = 0.89) and spiritual openness (α = 0.80).

According to Piedmont, spiritual transcendence is a feature shared by people around the world.

He indicates that spiritual transcendence is the sixth factor of personality.

It includes prayer fulfillment (feeling of joy from contact with transcendent reality), universality (belief in the unitive nature of life), and connectedness (belief that one is part of a larger human reality, necessary for maintaining harmony).

Spiritual transcendence is strictly related to religiousness. 

Religiousness and spirituality have a strong positive correlation, although they are not identical [23].

**Figure 1:**
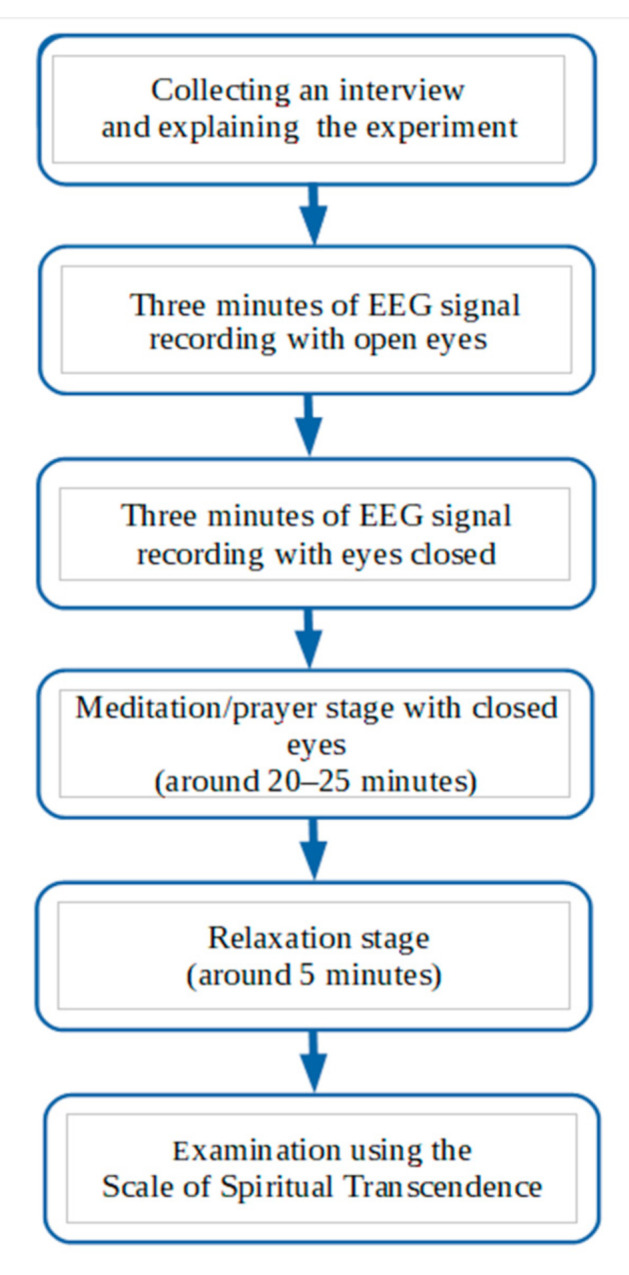
Stages of the experiment.

Both sub-scales consist of 11 questions with a possible score of 1 to 4 points each. Thus, the minimum and maximum possible score in the subscales is 11 and 44 points, respectively (22 and 88 points respectively for the entire scale). Full procedure on flowchart (Figure 1).

### 2.3. Statistical Analysis

The normality distribution was assessed using the Kolmogorov–Smirnov test (for all variables controlled in the study). The homogeneity of variance was verified using Levene’s test. The equality of covariance matrices was assessed using Box’s test. The obtained results allowed for applying parametric tests. Student’s *t*-test and the analysis of covariance (ANCOVA) were used to determine the significance of differences. Due to (only) two levels of independent variable measurement within each effect, no sphericity test was used in the covariance analysis. The significance level was set at *p* < 0.05. The effect size was assessed based on the partial η2 or Cohen’s d-factor. Data analysis was conducted in IBM SPSS Statistics 26 (IBM, Armonk, NY, USA).

## 3. Results

The mean, standard deviations, as well as the significance of differences (meditation/prayer vs. relaxation) with regard to the electrophysiological activity of the brain in individuals (EEG), transcendence, and spiritual openness (Buddhist meditators vs. Christian meditators) are presented in Table 1. Buddhist meditators demonstrated a statistically significantly higher theta amplitude at the Cz site during meditation compared to relaxation *(d* = 0.57). Other comparisons proved to be statistically insignificant.

**Figure 2:**
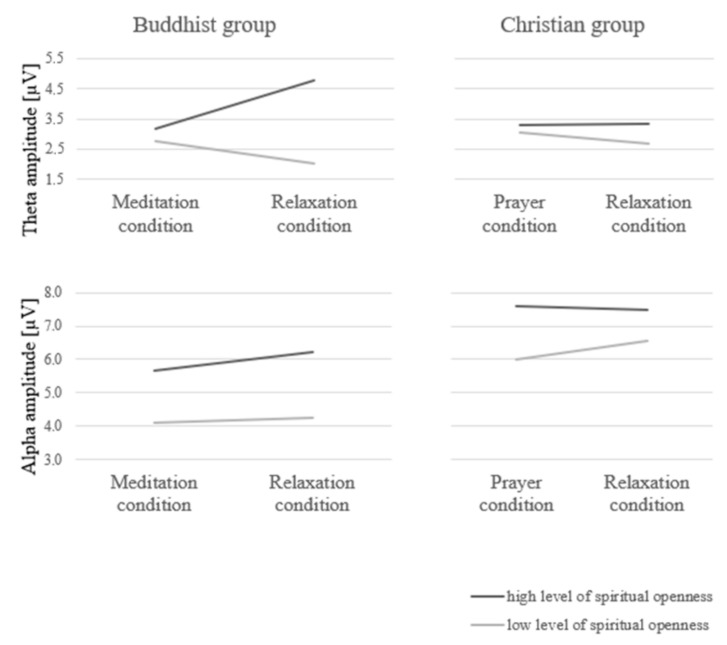
Theta and alpha amplitude at Pz (as per the 10–20 system) in the Buddhist and Christian groups in conditions of high and low level of spiritual openness.

In order to test the hypotheses, a 2 (group: Buddhists meditators vs. Christian-based faith) × 2 (measurement level: meditation/prayer vs. relaxation) covariate mixed ANCOVA was conducted. The covariates included: transcendence and spiritual openness. As a result of the analyses, no statistically significant main effects were obtained for the group and measurement level factor with regard to theta and alpha amplitudes in all of the controlled sites. As a result of the analyses, a statistically significant interaction effect was obtained for the group (Buddhists vs. Christians) × measurement level (meditation/prayer vs. relaxation) × spiritual openness factors in the theta amplitude range at Pz, *F* (2.17) = 6.61, *p* = 0.008, *η2* = 0.44. Analysis of simple effects showed that spiritual openness favored a higher theta amplitude at Pz during relaxation in Buddhists *(B* = 0.46, *SE* = 0.14, *t* = 3.36, *p* = 0.004). The significant interaction effect is presented in Figure 2 (the severity of the alpha amplitude was also presented for comparison). The remaining interaction effects proved to be statistically insignificant.

Theta amplitude [µV] M (SD) 1. Buddhist group: a. high level of spiritual openness—meditation condition = 3.18 (0.98), relaxation condition = 4.78 (2.04); b. low level of spiritual openness—meditation condition = 2.76 (0.66), relaxation condition = 2.04 (1.01); 2. Christian group: a. high level of spiritual openness—prayer condition = 3.30 (1.55), relaxation condition = 3.33 (1.02); b. low level of spiritual openness—meditation condition = 3.05 (0.94), relaxation condition = 2.67 (0.92); Alpha amplitude [µV] M (SD) 1. Buddhist group: a. high level of spiritual openness—meditation condition = 5.66 (2.36), relaxation condition = 6.20 (4.09); b. low level of spiritual openness—meditation condition = 4.10 (2.31), relaxation condition = 4.26 (2.05); 2. Christian group: a. high level of spiritual openness—prayer condition = 7.55 (4.91), relaxation condition = 7.53 (4.39); b. low level of spiritual openness—meditation condition = 6.01 (4.97), relaxation condition = 6.59 (4.35).

## 4. Discussion

In our study, Buddhist meditators exhibited a statistically significantly higher theta amplitude at Cz during meditation compared to relaxation. Spiritual openness in them favored a higher theta amplitude at Pz during relaxation. Our study did not indicate significant differences in frontal areas which were often indicated in previous studies. 

Researchers differ in their opinions as to which frequency is most characteristic and most relevant for the state of meditation/prayer.

When comparing the electrophysiological activity of meditation and relaxation, Lagopoulos revealed elevated values of alpha (in the posterior region) and theta (in the frontal and temporal-central regions) during meditation. Meanwhile, our analysis exhibited only an increase in the theta value at site Cz during meditation. These differences may stem from the different instructions that were given at the relaxation stage. Lagapoulos compared brain activity during meditation to a state of full relaxation understood as complete inactivity. In contrast, our study took meditation as a state of relaxation with a peaceful focus of one’s thoughts (on past, pleasant experiences). In this state, no strong emotions are evoked and the attention processes are not engaged that much, yet they are still targeted. In other words, in this context brain activity in our study was deprived of elements of spirituality [14]. Faber’s work also refers to comparing meditation with relaxation understood as inactivity. This difference may explain why we do not exhibit differences in alpha and theta activity, reported by the author [10].

Takahashi et al. revealed that during meditation there occurs an increase of theta high-frequency power and alpha low-frequency power in frontal brain areas, not observed by us [7]. However, also here we notice considerable methodological differences. The first one is the aforementioned difference in the relaxation stage. Moreover, in Takahashi, all participants came in contact with meditation for the first time. The last difference involves the manner of meditation, based on breath control [27].

Meanwhile, Aftanas indicated differences in alpha and theta increases during meditation among people with and without practicing experience. We could not make such observations due to an insufficient number of group members [6].

Our results are an interesting complementation of these reports. Under the conditions we created, where prayer/meditation was compared to targeted relaxation, we found no significant differences between groups regarding the alpha and theta bands. Such differences could be expected if these were processes related to open monitoring. This does not preclude the possibility of stating changes in the gamma band, which, however, need to be commented on with the objections raised in the introduction. Such observations were also made with regard to the prayers of Islam followers [28].

An interesting observation involves a higher theta during Buddhist meditation at Pz together with a high level of spiritual openness. The power of the theta band increases together with an increase of requirements pertaining to the tasks and is related to orientation, attention, memory, and affective processing mechanisms. Following Walker et al., electrophysiological activity from site Pz may be linked to cognitive processing, praxis, and spatial functions [29].

Spiritual openness, in turn, includes positively valued aspects of spirituality: respect for others, satisfaction with what life brings, and tolerance of ambiguity. This aspect seems more stressed in the Buddhist current, although no obvious link can be noticed here.

We believe that more research on this topic is necessary. In our opinion, meditation tasks should be referred not to inactivity, but to similar brain activity, yet without an element of spirituality. We believe that future studies should be expanded by a placebo group and individual alpha activity.

## 5. Conclusions

In our study, Buddhist meditators exhibited a statistically significantly higher theta amplitude at Cz during meditation compared to relaxation. Spiritual openness in them favored a higher theta amplitude at Pz during relaxation. Our study did not indicate significant differences in frontal areas which were often indicated in previous studies.

## Figures and Tables

**Table 1:** Mean values regarding EEG, transcendence, and spiritual openness in participants.

Electrophysiological Brain Activity in Participants [µV]							
Buddhist Meditators (*N* = 10)							
	**Meditation**		**Relaxation**		**t(9)**	***p***	**d**
	M	SD	M	SD			
Theta Fp1	6.98	2.50	6.81	3.39	0.29	0.777	0.06
Theta Fp2	6.81	2.86	6.57	2.12	0.25	0.806	0.10
Theta Fz	5.93	1.65	6.04	1.57	−0.17	0.871	−0.07
Theta Cz	4.07	1.18	3.53	0.65	2.33	0.045	0.57
Theta Pz	2.97	0.80	3.41	2.46	−0.55	0.594	−0.24
Theta O1	4.98	1.69	4.00	0.90	1.68	0.126	0.72
Theta O2	3.73	1.60	2.77	1.55	2.22	0.054	0.61
Alpha Fp1	10.99	5.18	9.28	4.38	1.59	0.147	0.36
Alpha Fp2	11.07	5.41	9.02	4.36	1.67	0.130	0.42
Alpha Fz	10.19	5.36	8.66	3.80	1.27	0.235	0.33
Alpha Cz	8.75	5.40	7.73	4.54	1.04	0.326	0.20
Alpha Pz	4.88	2.35	5.23	3.22	−0.58	0.575	−0.12
Alpha O1	5.78	2.76	5.12	2.75	1.83	0.101	0.24
Alpha O2	3.72	1.57	3.59	1.70	0.48	0.641	0.08
**Christian-Based Faith (*N* = 12)**							
	**Prayer**		**Relaxation**		**t(11)**	***p***	**d**
	M	SD	M	SD			
Theta Fp1	6.37	1.64	6.01	1.62	0.80	0.439	0.22
Theta Fp2	6.50	1.68	6.47	1.48	0.05	0.965	0.02
Theta Fz	7.38	2.62	6.90	1.45	0.57	0.578	0.23
Theta Cz	4.18	1.27	4.17	1.09	0.05	0.965	0.01
Theta Pz	3.18	1.23	3.00	0.98	0.77	0.458	0.16
Theta O1	6.24	2.09	5.28	0.58	1.59	0.139	0.63
Theta O2	2.92	1.22	3.62	2.96	−1.02	0.332	−0.31
Alpha Fp1	12.67	8.24	11.96	7.88	0.83	0.426	0.09
Alpha Fp2	12.67	8.08	12.09	7.85	0.67	0.517	0.07
Alpha Fz	12.89	8.75	12.19	7.78	0.89	0.393	0.08
Alpha Cz	10.93	8.11	10.16	7.08	1.11	0.290	0.10
Alpha Pz	6.78	4.84	7.06	5.21	−0.47	0.647	−0.06
Alpha O1	6.74	3.47	6.38	4.05	0.80	0.443	0.10
Alpha O2	4.18	2.58	4.34	2.40	−0.57	0.579	−0.06
**Psychological Variables**							
	**Buddhist meditators**		**Christian-based faith**		**t(20)**	***p***	**d**
	M	SD	M	SD			
Transcendence	36.90	6.56	37.08	4.74	−0.08	0.940	−0.03
Spiritual openness	37.80	4.05	36.17	1.95	1.17	0.265	0.51
